# What clinicians who practice in countries reaching malaria elimination should be aware of: lessons learnt from recent experience in Sri Lanka

**DOI:** 10.1186/1475-2875-10-302

**Published:** 2011-10-14

**Authors:** Ranjan Premaratna, Gowrie Galappaththy, Nilmini Chandrasena, Roshanthi Fernando, Thusha Nawasiwatte, Nilanthi R de Silva, H Janaka de Silva

**Affiliations:** 1 Department of Medicine, Faculty of Medicine, University of Kelaniya, Colombo, Sri Lanka; 2 Anti-Malaria Campaign, Colombo, Sri Lanka; 3 Department of Parasitology, Faculty of Medicine, University of Kelaniya, Colombo, Sri Lanka

## Abstract

Following progressive reduction in confirmed cases of malaria from 2002 to 2007 (41,411 cases in 2002, 10,510 cases in 2003, 3,720 cases in 2004, 1,640 cases in 2005, 591 cases in 2006, and 198 cases in 2007). Sri Lanka entered the pre-elimination stage of malaria in 2008. One case of indigenous malaria and four other cases of imported malaria are highlighted here, as the only patients who presented to the Professorial Medical Unit, Colombo North Teaching Hospital, Ragama, Sri Lanka over the past eight years, in contrast to treating several patients a week about a decade ago. Therefore, at the eve of elimination of malaria from Sri Lanka, it is likely that the infection is mostly encountered among travellers who return from endemic areas, or among the military who serve in un-cleared areas of Northern Sri Lanka. They may act as potential sources of introducing malaria as until malaria eradication is carried out. These cases highlight that change in the symptomatology, forgetfulness regarding malaria as a cause of acute febrile illness and deterioration of the competency of microscopists as a consequence of the low disease incidence, which are all likely to contribute to the delay in the diagnosis. The importance regarding awareness of new malaria treatment regimens, treatment under direct observation, prompt notification of suspected or diagnosed cases of malaria and avoiding blind use of anti-malarials are among the other responsibilities expected of all clinicians who manage patients in countries reaching malaria elimination.

## Background

The history of malaria in Sri Lanka dates back to the ancient kingdoms. During the long documented history of malaria in Sri Lanka several major epidemics were experienced. The most devastating of these was the epidemic of 1934 - 1935 during which the districts in the wet zone and the intermediate zone experienced high incidence resulting in nearly 1.5 million patients and 80,000 deaths. In the last two decades, major epidemics were encountered during the years 1987 and 1990/92. Major natural determinants of malaria epidemics in Sri Lanka have been the monsoon rains especially the North East monsoon, and also unusually dry weather leading to pool formation in rivers and streams [1,2]. Following the introduction of dichloro-diphenyl-trichloroethane (DDT) for mosquito vector control, there was a record reduction in the cases of malaria, with just seventeen cases in 1963 [2,3]. A similar scenario was experienced in Crete after the introduction of DDT in 1939 reduced malaria cases to undetectable levels within five years, enabling discontinuation of spraying DDT and declining malaria control activities [4]. However, within a matter of few years there was a resurgence of malaria in Crete due to the emergence of DDT resistant vector species [4,5]. Sri Lanka also experienced a resurgence of malaria in about five years. A massive malaria epidemic was experienced during the years 1967 - 1969 due to relaxation of surveillance. Although the exact mechanism is not known, several factors were thought to be contributory towards the failure. Persistence of several undetected foci of malaria transmission, extensive intra-country population movements particularly related to gem mining, lack of adequate financial support from the authorities at the time when the incidence was extremely low are thought to have contributed to this [2]. Since then, malaria became entrenched as a major illness in the island causing several hundreds of thousands of cases each year and many deaths.

**Figure 1:**
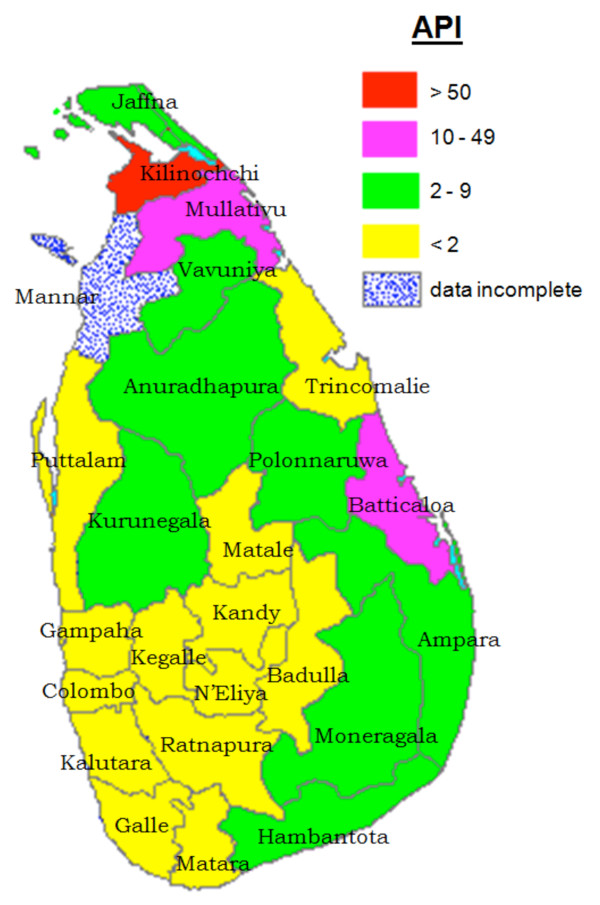
**Annual Parasite Incidence (API) (total number of positives cases per 1000 risk population) for the Year 2002**.

**Figure 2:**
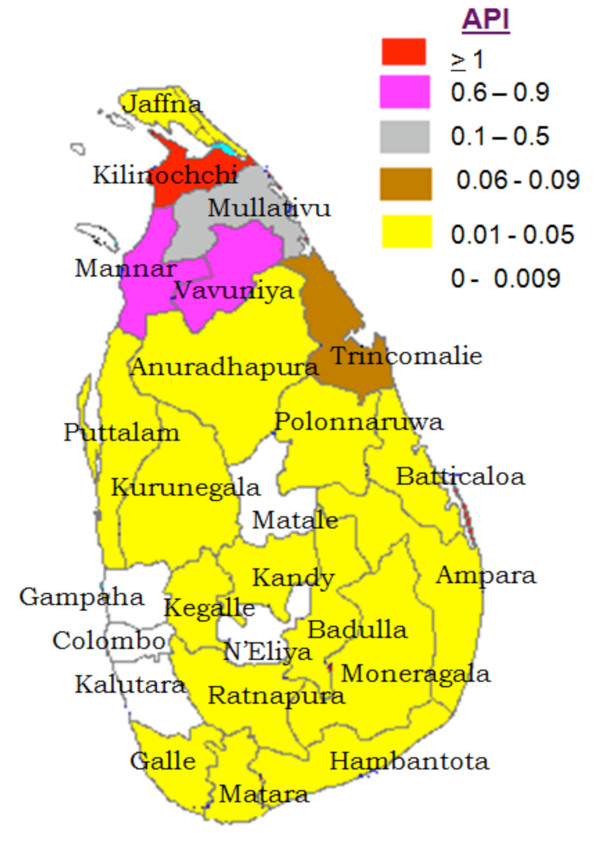
**Annual Parasite Incidence (API) (total number of positives cases per 1000 risk population) for the Year 2008**.

**Figure 3:**
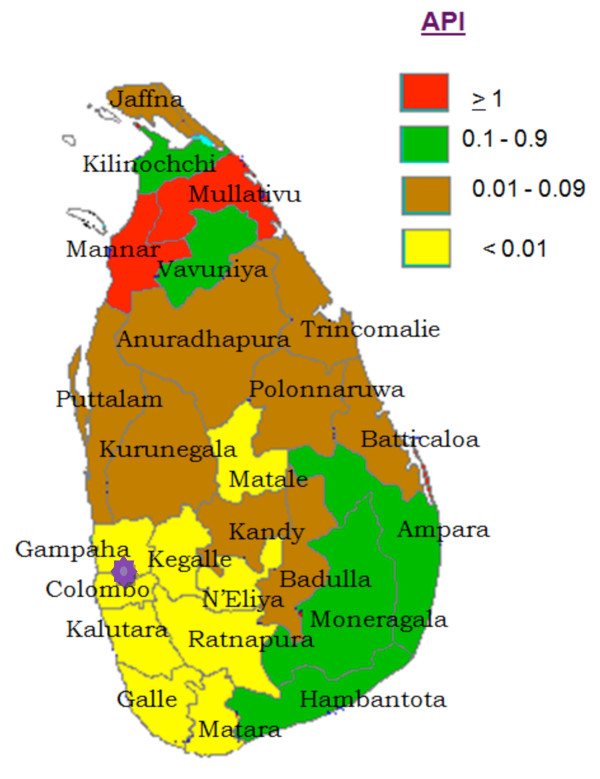
**Annual Parasite Incidence (API) (total number of positives cases per 1000 risk population) for the Year 2010**. The star shows the location of Colombo North Teaching Hospital, Ragama. All the patients were residing within 25 Km from the hospital

The Anti-Malaria Campaign (AMC) in Sri Lanka which continued on eradication principals for several years was subsequently, reoriented as a control program which included many elements of the earlier eradication programme. During the past two decades or more, the AMC had been functioning as a control programme geared at achieving set objectives. Operationally, the AMC had a centralized structure till 1989 and functioned as a vertically run programme. However, in 1989 the programme was transformed into a decentralized campaign, which was implemented by nine provincial programs under the technical guidance of National AMC Directorate. Due to efficient activities of AMC [2], together with improvement in standards of housing in populations in endemic regions and the use of mosquito nets; especially those impregnated with insecticide, by early 2000, there was a significant reduction in malaria cases in the southern parts of the island. The confirmed cases of malaria [Total (Vivax: Falciparum)] from 2002 to 2007 were; 41,411 cases in in 2002 (36,563:4,848), 10,510 cases in 2003-(9,237:1,273), 3,720 cases in 2004 (3,171:5,49), 1640 cases in 2005 (1,506:134), 591 cases in 2006 (564:27), 198 cases in 2007 (191:7) [6-8]. Meanwhile, malaria remained a problem in the northern and eastern regions of the country due to the civil unrest. However, despite the ongoing conflict, the AMC managed to deliver its services effectively, even in the deep jungles, with the support of all district or provincial malaria programmes and a few NGOs. In 2006 and 2007, the reported malaria cases in the country dropped to 591 and 198 respectively [7,8]. Therefore, in 2008, Sri Lanka entered the pre-elimination phase of malaria control, together with nine other countries: Mexico, Iran, Azerbaijan, Georgia, Kyrgyzstan, Tajikistan, Turkey, Uzbekistan and Democratic Republic of Korea [9]. Annual Parasite Incidence (API) (total number of positives cases per 1,000 risk population) for the Year 2002, 2008 and 2010 are presented in Figures 1, 2 and 3.

The following cases of malaria, all seen at the Colombo North Teaching Hospital, Ragama, Sri Lanka since 2003, illustrate the problems that are likely to be encountered during attempts to eliminate malaria.

## Case presentations

### Case 1

In November 2007, a soldier, who was serving in an area which were previously affected by the 30 years of civil unrest in Jaffna, in northern Sri Lanka, presented with fever and body aches after arriving home in the Colombo suburbs; an area which is now endemic for dengue. He was suspected to be suffering from dengue fever, but was detected to be having *Plasmodium vivax *malaria on a routine blood film. He was successfully treated with chloroquine and a 14-day course of primaquine, ensuring clearance of malaria parasites by repeated parasite counts (Table 1). The disease was notified immediately to the regional anti-malaria office which enabled detection of a mini-outbreak of malaria in Jaffna and among few of his colleagues who returned to Hambantota, a southern city which was endemic for malaria in the past. All these patients were rapidly detected and treated, and it is possible that re-transmission of malaria was curtailed.

**Table 1:** Patient details

Case	Age	Sex	Admitted	Clinical	Haematology	Biochemistry	MP	Treatment	country visited
1	46 y	M	November 2007	High intermittent fever, Body aches, vomiting, diarrhoea of 4 days, 2 cm splenomegaly and tender hepatomegaly 3 cm	Hb-11 g/dL, WBC-4.8 × 10^9^/L N-55%, Platelet 110 × 10^9^/L	ALT-110 iu/L, AST-140 iu/L	*P. vivax *Parasite count 3760/μL	Chloroquine Premaquine	None

2	18 y	M	April 2008	High intermittent fever, severe headache, vomiting, 4 days; progressed to confusion, rigidity over 24 hours, 3 cm hepatomegaly	Hb-10 g/dL, WBC-5.6 × 10^9^/L, N-64%, Platelet 158 × 10^9^/L	ALT-90 iu/L AST-58 iu/LB. Urea: 65 mg/dL	*P. malariae *and *P falciparum *parasite count 26,000/μL	iv Quinine + Doxycycline+ Co artum + Premaquine	Johannesburg South Africa

3	33 y	M	1^st ^admission: December 2009	Shortness of breath on exertion, lethargy for one month, sever pallor, 6 cm Splenomegaly	Hb- 5.5 g/dL, WBC 6.2 × 10^9^/L, Platelets-230 × 10^9^/L, Basophilic stippling in red cells	ALT- 56 iu/L, AST-110 iu/L, Serum Pb > 65 mg/dL	Malaria parasite nil. Malaria Ag negative	Penicilamine	Uganda

		M	2^nd ^admission: January 2010	High intermittent fever, sever pallor, ill and toxic, 8 cm Splenomegaly and 4 cm hepatomegaly	Hb-6.5 g/dL, WBC-2.2 × 10^9^/L, Platelets-65 × 10^9^/L, Retic count-5% Bone marrow biopsy: normal	SGOT-100 iu/l, SGPT-248 iu/L S.Bilirubin-normal S.Protein-5.1 g/dl, Alb-3.7 g/dl, Glob-1.3 g/dl γGT-69.2 iu/L PT & INR-1.14	*P. falciparum*Parasite count 3550/μL	Iv Quinine + Co artum + Premaquine	

4	35 y	M	September-2010	High intermittent fever, mild icterus, body aches, headache, vomiting, 2 cm splenomegaly, 4 cm hepatomegaly	Hb-10.5 g/dL, WBC-2.8 × 10^9^/L, Platelets-45 × 10^9^/L,	ALT-430 iu/L, AST-560 iu/L, S.Bil 3.4 mg/dl B. Urea: 98 mg/dL	*P. vivax + ve *(serology for Dengue leptospira, Viral hepatitis A, B, C, E negative)	Chloroquine + Premaquine	Kolkata India

5	28 y	M	October 2010	High intermittent fever, mild icterus, body aches, headache, vomiting, 4 cm splenomegaly, 3 cm tender hepatomegaly	Hb-11.5 g/dL, WBC-6.2 × 10^9^/L, Platelets-35 × 10^9^/L,	ALT-630 iu/L, AST-1054 iu/L, Serum Bilirubin 2.4 mg/dl B. Urea: 45 mg/dL			

### Case 2

**Figure 4:**
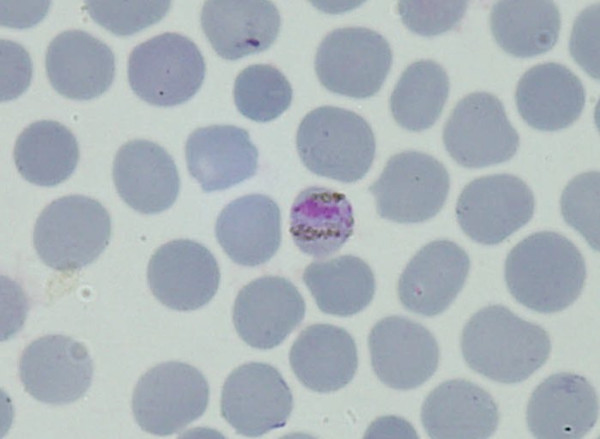
***Plasmodium malariae *band form in patient 2**.

In April 2008, an 18-year-old Sri Lankan male, who had returned from Johannesburg South Africa three weeks before, presented with fever, headache and confusion over three days. He rapidly developed opisthotonus (Table 1). At the very onset, he was suspected to be having virus encephalitis. A blood film examined in a private laboratory showed *P. vivax *ring forms, which was considered not compatible with his clinical illness. However a thin blood smear reported by a specialist detected *Plasmodium malariae *(Figure 4) and *Plasmodium falciparum *mixed infection. The patient was successfully treated with intravenous quinine followed by doxycycline and Co-Artem^® ^as advised by the AMC.

### Case 3

**Figure 5:**
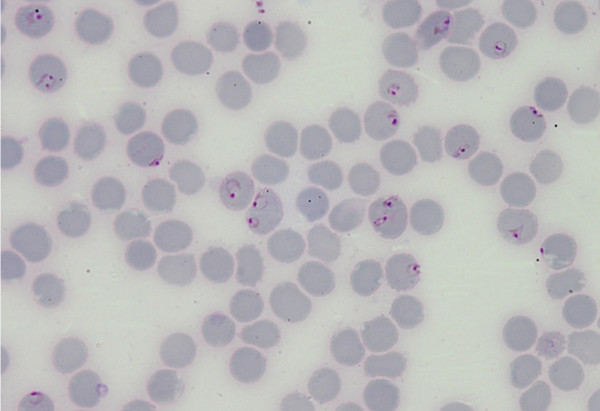
***Plasmodium falciparum *ring forms in patient 3**.

In December 2009, a 36-year-old Sri Lankan male employed in Uganda presented with severe pallor and 5 cm firm splenomegaly (Table 1). He had returned to the island about a month before due to feeling "lethargic". He had repeatedly received short courses of the anti-malarial "amodiaquine" while he was in Uganda for non-specific aches and pains. His haemoglobin on admission was 5.5 g/dl and blood tests were negative for malaria parasites and malaria antigens. His blood picture showed basophilic stippling of red blood cells and he was subsequently detected having high serum lead levels confirming lead poisoning. He was commenced on oral penicillamine and received four units of packed-red-cell transfusion for anaemia. He was re-admitted with high fever after one month and had severe pancytopaenia and was suspected to have penicillamine toxicity. He was commenced on intravenous antibiotics, but did not show clinical improvement over the next few days. A repeat blood film showed *P. falciparum *ring forms (Figure 5) and *P. falciparum *antigen was positive. He was successfully treated with i.v. quinine followed by Co-Artem^®^.

### Cases 4 and 5

In September - October 2010, two Sri Lankan patients both who returned home from Kolkata India presented with high fever and severe body aches for four days (Table 1). Investigations revealed progressive lowering of platelets counts and rising hepatic enzymes, suggesting dengue fever. Dengue and Leptospira antibodies were negative. They were detected to have *P. vivax *malaria on routine blood films and responded to anti-malarials chloroquine followed by 14-day primaquine.

## Consent

Consent was obtained from all relevant patients to include their clinical details.

## Ethical permission

Ethical permission to publish clinical details of patient was obtained from the Ethics

Review committee, Faculty of Medicine, University of Kelaniya, Sri Lanka

## Discussion

The only five cases of malaria who presented to our unit since 2003 are presented here. None of them had past history of malaria. They were living within 25 km of the hospital (Figure 3). The first case was unexpected in southern Sri Lanka which is now considered non-endemic for malaria. The patient was diagnosed to have malaria only accidently as malaria is not considered a common cause of short duration febrile illness in these areas. The other cases were imported malaria with varying degrees of severity and posing diagnostic dilemmas due to the considerable time interval between return to Sri Lanka and clinical presentation, their clinical presentations suggesting an alternative diagnosis or due to erroneous interpretation of blood films.

Occurrence of malaria in most southern parts of the country is very rare during the last several years. Therefore, clinicians practicing in these areas have almost forgotten it as a causative agent for short duration febrile illness. However, they should be aware of the possibility of unidentified asymptomatic low-grade parasitaemia or occurrence of clinical *P. vivax *malaria due to relapse that may contribute to individual cases or mini-outbreaks until malaria eradication is achieved. Although, a recent study failed to demonstrate detectable antigenaemia within these populations, in order to promptly diagnose such patients and also to achieve the final objective of elimination of malaria, continued surveillance, prompt parasitological confirmation and strict avoidance of blind treatment with anti-malarials is required [10,11]. Furthermore, if malaria occurs following blood transfusions, blood donors from endemic areas should be re-checked to ensure that they are free from asymptomatic malaria as they could function as potential sources for future outbreaks in endemic areas. The individuals living within 1 km of an index case or a potential breeding site are known to be at risk of malaria [12]. Therefore, vigilance is needed for any febrile patient who lives within this geographical boundary. Recent studies on anopheles breeding sites has demonstrated breeding of major Anopheles vector species that transmit malaria in brackish water collections in Sri Lanka [13] making any geographical location a vulnerable site for vector breeding.

Except for the first patient all the other four patients had returned to the country from a malaria endemic region and they presented from April 2008 - October 2010. They were the only patients with malaria who were admitted to our medical unit from the beginning of 2003 to end of 2010, compared to ten or more patients with indigenous malaria admitted a month prior to 2000. Similar to in 1963, when the lowest ever malaria incidence of seventeen cases were recorded and, eleven out of the seventeen were considered imported cases [3], today, the occurrence of malaria seems to be a consequence of expanding travel outside the country. Foreign employment and participation in long duration training or study courses in malaria endemic areas and UN missions by Sri Lankan security personnel account for most cases of imported malaria. Although short duration travel carries a low risk, such travel to malaria endemic countries has resulted in infections. Pitfalls in the diagnosis of imported malaria could occur due to delayed symptoms, especially among those who return after long stays in endemic countries as observed in the 2^nd ^and 3^rd ^cases; this could either be due to weaning of immunity following absence of repeated exposure to malaria after return [14,15], or suppression of a partially resistant strain of *P. falciparum *to repeated doses of amodiaquine given as prophylaxis or as part of treatment in an endemic setting [16]. Altered symptomatology, occurrence of more severe forms of illness due to vivax malaria, liver and renal involvement, and reduction in platelet counts [17-19] may lead to erroneous diagnoses such as dengue fever or leptospirosis, as observed in the 4^th ^and 5^th ^cases. Waning of skills and experience of microscopists (2^nd ^and 3^th ^cases) due to the low incidence of malaria may also contribute to a delay in diagnosis [20].

In the face of the problem of erroneous microscopic diagnosis by technicians, it is mandatory that any suspected or "diagnosed" case be re-confirmed by a specialist and/or by detecting antigens through rapid diagnostic kits, before they are commenced on treatment. Any doubts in the diagnosis should be clarified by the WHO reference laboratory by genotyping.

Clinicians should adhere to treatments recommended for the elimination phase and in the event of imported malaria, they should liaise with the AMC or relevant authorities for the most appropriate treatment for such patients, as was done in case 2.

## Conclusions

Sri Lanka entered the pre-elimination stage of malaria in 2008. Infection is mostly encountered among travellers who return from endemic areas such as African countries and from India, or among military personnel serving in the northeast of the country. They may act as potential sources of reintroducing malaria, as elimination of the vector is not feasible. The change in symptomatology, forgetfulness of malaria as a cause of acute febrile illness and deterioration of the competence of microscopists are all likely to contribute to a delay in the diagnosis. Awareness of new treatment regimens, treatment under direct observation and prompt notification of suspected/diagnosed cases to the AMC are the responsibility of all clinicians who manage patients with malaria. Therefore, a good step-wise surveillance programme to detect all malaria cases either indigenous or imported, will help achieving the final goal of eliminating malaria from Sri Lanka.

## Competing interests

The authors declare that they have no competing interests.

## Authors' contributions

RP, RF, TN, JDES participated in active management of all patients, RP, RF, TN recorded and retrieved clinical data. GG, NC, NDES carried out parasitological identification and confirmation, GG advised on active management of patients and provided the maps to highlight malaria incidence in Sri Lanka, RP, GG, NC, NDES and JDES wrote up the manuscript. All authors read and approved the final version of the manuscript.
